# Association of primary care physician supply with maternal and child health in China: a national panel dataset, 2012–2017

**DOI:** 10.1186/s12889-020-09220-4

**Published:** 2020-07-11

**Authors:** Mengping Zhou, Luwen Zhang, Nan Hu, Li Kuang

**Affiliations:** 1 Department of Health Administration, School of Public Health, Sun Yat-sen University, No.74, Zhong Shan Er Road, Guangzhou, 510080 China; 2 Department of Health Management, School of Health Services Management, Southern Medical University, Guangzhou, 510515 China; 3 Department of Biostatistics, FIU Robert Stempel College of Public Health and Social Work, Miami, FL 33199 USA; 4 Department of Family and Preventive Medicine, and Population Health Sciences, University of Utah School of Medicine, Salt Lake City, UT 84132 USA

**Keywords:** Primary care physician supply, Maternal and child health outcomes, Health disparities

## Abstract

**Background:**

The Chinese government has been strengthening the primary care system since the launch of the New Healthcare System Reform in 2009. Among all endeavors, the most obvious and significant improvement lays in maternal and child health. This study was designed to explore the association of primary care physician supply with maternal and child health outcomes in China, and provide policy suggestions to the law makers.

**Methods:**

Six-year panel dataset of 31 provinces in China from 2012 to 2017 was used to conduct the longitudinal ecological study. Linear fixed effects regression model was applied to explore the association of primary care physician supply with the metrics of maternal and child health outcomes while controlling for specialty care physician supply and socio-economic covariates. Stratified analysis was used to test whether this association varies across different regions in China.

**Results:**

The number of primary care physicians per 10,000 population increased from 15.56 (95% CI: 13.66 to 17.47) to 16.08 (95% CI: 13.86 to 18.29) from 2012 to 2017. The increase of one primary care physician per 10,000 population was associated with 5.26 reduction in maternal mortality per 100,000 live births (95% CI: − 6.745 to − 3.774), 0.106% (95% CI: − 0.189 to − 0.023) decrease in low birth weight, and 0.419 decline (95% CI: − 0.564 to − 0.273) in perinatal mortality per 1000 live births while other variables were held constant. The association was particularly prominent in the less-developed western China compared to the developed eastern and central China.

**Conclusion:**

The sufficient supply of primary care physician was associated with improved maternal and child health outcomes in China, especially in the less-developed western region. Policies on effective and proportional allocation of resources should be made and conducted to strengthen primary care system and eliminate geographical disparities.

## Background

Considerable evidences have proven internationally that primary care strengthening is the most cost-effective way to improve the health outcomes, and most countries are determined to establish health care system oriented to primary care [1]. The increased supply of primary care physician and high-quality primary care were confirmed to have health-promoting influence, regardless of various health outcomes (all-cause mortality, cause-specific mortality, infant mortality, and life expectancy), the level of analysis (cross-sectional or longitudinal), or the units of analysis (international, state, county, or local area) [2–5].

Accumulating evidences also show the considerable potential contribution of a strong primary care system to reduce racial and income related health disparities and provide better protection for vulnerable populations [6–10], and the strength of primary care system is usually measured by the supply of physicians [2], the core functions of primary care [11], and patient perceived primary care quality [12]. A literature review which has summarized most of US studies highlighted the positive effects of increased primary care physician supply on more equitable health service distribution, improved overall health and reduced gaps in health across major population groups [5]. It has indicated that the core functions of primary care, namely, continuity, comprehensiveness, accessibility, coordination of care and community-based services [11], are accountable for the beneficial health impacts [5, 13]. A series of WHO reports support as well that primary care is the first choice to bridge availability gap, achieve universal coverage and eliminate health disparities among regions and subpopulations [14].

The establish of primary care system in China started in the 1950s, and contributed to the control of communicable diseases and the improvement of public health [15]. The barefoot doctors who received minimal basic medical and paramedical training acted as primary health care providers and worked in villages to offer universal primary care services at very low price [16]. This primary care provision model soon increased China’s life expectancy from 35 in 1949 to 65 in the early 1980s [17] and made prototype for the world’s primary care system. Though the primary-care-oriented system was abandoned nationally after the Reform and Opening-up in the 1980s, the 2009 New Healthcare System Reform returned to the track of strengthening primary care system [18]. Large amount of government subsidies—increased from CNY 19.8 billion (USD 2.8 billion) in 2008 to CNY 197.7 billion (USD 27.8 billion) in 2018 [19]—were directed to rebuild the primary care delivery system with main efforts of promoting the standardized construction of basic infrastructures, training the generalists with the new “5 + 3” education programs, and implementing the universal service standards [18, 20]. Meanwhile, universal health insurance coverage, national essential drug system and Basic Public Health Services (BPHS) program were implemented to improve the accessibility to and affordability of primary care [21]. BPHS, covering 14 basic public health service items, was provided by primary care physicians to all Chinese residents for free, and maternal and child health management services was one of them [22].

Maternal and child health (MCH), a major concern of global health and priority of MDGs and SDGs, have been widely used to measure the social and economic development, national health status and social equity [23]. In the past two decades, MCH have greatly improved in China [24, 25]: maternal mortality rate dropped from 53.0/100,000 in 2005 to 19.6/100,000 in 2017, neonatal mortality rate decreased from 13.2/1000 in 2005 to 4.5/1000 in 2017, and the disparity between rural and urban narrowed from 2 to 3 times in 2005 to 1–2 times in 2017. However, the substantial geographical inequities of MCH outcomes still exist in China [26].

Primary care system benefits the health of mothers, newborns and children. Starfield and Shi found that the states with higher ratio of primary care physicians to population in US was associated with lower infant mortality and fewer low birth weight after controlling for sociodemographic measures [27]. In Brazil, after the government made interventions to remove obstacles of accessibility to primary care, the maternal and infant mortality rates reduced substantially by 4.4% a year since 2000, and the regional and socioeconomic inequalities in access to such interventions were notably reduced [28]. Bhutta et al. made a systematic review of the evidence-based intervention cases in Pakistan and Uganda and proved that primary health care at pragmatic coverage in these two countries could prevent 20–30% of all maternal deaths, 20–21% of newborn deaths, and 29–40% of all post-neonatal deaths in children aged less than 5 years [29]. There are diverse evidences on the contribution of primary care to MCH outcomes in developed and developing countries, but few studies on the progresses in China [30]. It is worthy to explore and evaluate the contribution of primary care to MCH improvement in China. Previous researches used different analytic approaches based on the data or study design to confirm the potential beneficial impact of PCP supply on improving health outcomes, such as Poisson regression models for counting data [31], linear random-effects panel data model for rural county-level data from 2014 to 2016 [32], and mixed-model method for 11 years of US state level data [27].

This study used provincial level data from 2012 to 2017 with a linear regression panel data model to investigate the association of PCP supply with the metrics of MCH outcomes. We also tested whether this association varies across the eastern, central, and western regions by stratified analysis to explore if the supply of primary care physician can reduce geographical health disparities in China.

## Methods

### Study design

The study was a longitudinal ecological analysis based on 6-year balanced panel data of 31 provinces in mainland China (excluding Hong Kong, Macau and Taiwan). Province was set as the basic analysis unit to reduce the likelihood of random fluctuations in mortality and population size, and attenuate the probable crossover effect when measuring availability of medical care and mortality in the smaller units of analysis like cities or counties [33]. Panel data was advantageous in increasing the estimation of precision and accounting for individual heterogeneity due to the effectiveness in controlling for unmeasurable factors influencing health outcomes and composing a larger sample size over time periods.

### Data source and variable

MCH outcomes was measured by three indicators: (1) maternal mortality rate (MMR) to measure maternal health, defined as the number of maternal deaths per 100,000 women of reproductive age in the population; (2) low birth weight (LBW), defined as the percentage of infant weighed less than 2500 g in all live births and (3) perinatal mortality rate (PMR), defined as the sum of neonatal deaths and fetal deaths (stillbirths) per 1000 births, to measure child health.

The primary care physician supply, one of the most widely used indicators to measure the strength of primary care, was defined as the number of primary care physicians (PCPs) per 10,000 population at provincial level. PCPs refer to all licensed doctors and assistant doctors who work in primary care institutions, including community health centers, township health centers, outpatient clinics and village clinics according to National Health Statistics Center of China.

Since the MCH outcomes was also influenced by the specialty care system [34], the specialty care physicians supply, defined as the number of physicians working in secondary or tertiary hospitals per 10,000 population, was adopted as a structural indicator of the health care system. To control for the socio-economic covariates, GDP per capital, proportion of illiterate population aged ≥15, registered urban unemployment rate and penetration rate of sanitary toilets in rural areas were adopted as indicators of economic, education, occupation and hygiene status.

Data of education and occupation were obtained from National Bureau of Statistics, and the rest were obtained from “*China Statistical Yearbook on Health and Family Planning 2013-2018*”.

### Statistical analysis

We used a linear regression model for panel data to explore the association of PCP supply with the metrics of MCH outcomes. One of the two panel data models, namely fixed- or random-effects models, was selected during the analysis process. In this study, province was the basic analysis unit. There may be some unmeasurable and time-invariant factors influencing the MCH outcomes such as historical or cultural factors that are unique to each province and independent of other provincial characteristics, so the fixed-effects regression model may be more applicable. In addition, the Hausman test that determines which model to choose also indicated that fixed-effects model should be applied (*p* < 0.05) [35]. Year was also included in the model as a dummy variable to control for unmeasured time variant characteristics such as new developments in technology or changes in national health policies that would affect all provinces. The equation for the fixed-effects regression model can be written as:

$$ {Y}_{it}={\beta}_0\kern0.5em +\kern0.5em \beta {X}_{it}\kern0.5em +\kern0.5em \delta {T}_t\kern0.5em +\kern0.5em {a}_i\kern0.5em +\kern0.5em {\varepsilon}_{it} $$

where *i* (*i* = 1···,31) represents the province, *t* is number of years since 2012 (*t* = 0···,5), *Y*_*it*_ represents the dependent variable for province *i* at time *t*, ***X***_*it*_ represents the independent variable for province *i* at time *t*, *T*_*t*_ is a vector of year-dummies (have *t*-1 time periods). *β*_*0*_ is the mean intercept of the 31 provinces over the 6 years, *β* represents the influence coefficient of the independent variables on the dependent variables, *δ* is the coefficient for the year-dummies, *α*_*i*_ is the deviation of province-specific intercept from mean intercept *β*_*0*_, *ε*_*it*_ represents error disturbance term.

In the regression analyses, the predictor variables were added into the model for each MCH indicator step by step to assess changes in the direction and magnitude of coefficient for the primary care indicator. In model 1, only the PCP supply was included as independent variable. In model 2, specialized care physician supply was added. In model 3, other socio-economic covariates were included. Alongside, we performed a sensitivity test with 1 year lagged primary care indicator to test the robustness of results. We estimated the effect of a previous year’s PCPs per 10,000 population on this year’s MCH outcome.

In the stratified analyses, 31 provinces were divided into three regions—the eastern (11 provinces), central (8 provinces), and western (12 provinces). The process and method of regression analysis for the stratified regions were the same as the whole country. All analyses were conducted using Stata 14 software.

## Results

**Fig. 1:**
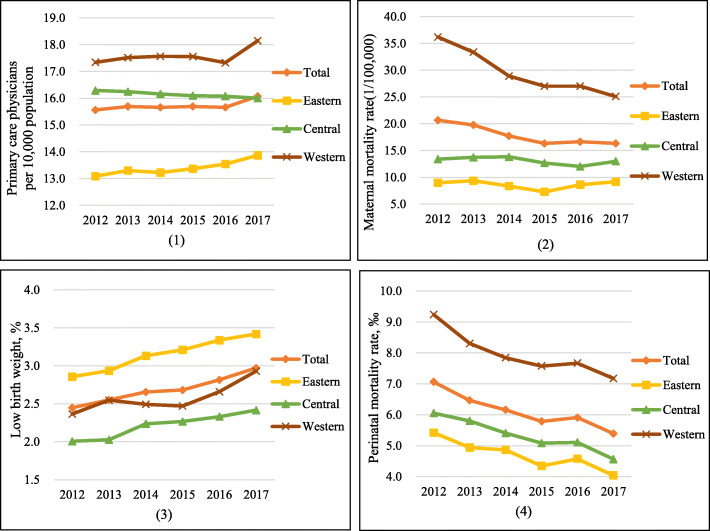
((1)–(4)) The changing trends for primary care indicator, maternal and child health indicators

Table 1 presented descriptive statistics of all variables from 2012 to 2017. The number of PCPs per 10,000 population was stable at around 15.6 except a sudden increase in 2017 while the supply of specialty care physician increased obviously from 10.90 to 14.47 per 10,000 population. Both MMR and PMR declined smoothly in 2012–2015 and then fluctuated in 2016–2017. In the same period, a smooth increase was found in the LBW, from about 2.45 to 2.97% of live births. The changing trends of primary care indicator and MCH indicators in the eastern, central, and western regions were basically consistent with that in the whole country from 2012 to 2017 (Fig. 1). The MMR and PMR in the western region were both apparently higher than that in the other two regions in each year. What’s more, the gaps of MMR/PMR between different regions narrowed somewhat but were still evident.

**Table 1:** Descriptive statistics of all variables in 31 provinces of China, 2012–2017

Variable	Mean (95% CI)					
	2012	2013	2014	2015	2016	2017
Primary care physicians per 10,000 population	15.56 (13.66 to 17.47)	15.69 (13.71 to 17.68)	15.66 (13.58 to 17.74)	15.69 (13.62 to 17.77)	15.66 (13.76 to 17.57)	16.08 (13.86 to 18.29)
Maternal mortality rate (1/100,000)	20.66 (9.59 to 31.73)	19.78 (10.13 to 29.44)	17.72 (10.83 to 24.61)	16.32 (9.89 to 22.74)	16.63 (9.80 to 23.46)	16.32 (10.38 to 22.26)
Low birth weight, %	2.45 (2.12 to 2.78)	2.55 (2.16 to 2.94)	2.65 (2.32 to 2.99)	2.68 (2.34 to 3.02)	2.82 (2.49 to 3.14)	2.97 (2.62 to 3.32)
Perinatal mortality rate, ‰	7.06 (5.61 to 8.51)	6.46 (5.28 to 7.65)	6.16 (5.08 to 7.23)	5.78 (4.67 to 6.90)	5.91 (4.79 to 7.03)	5.39 (4.34 to 6.43)
Specialty care physicians per 10,000 population	10.90 (9.73 to 12.06)	11.60 (10.47 to 12.73)	12.13 (10.99 to 13.28)	12.90 (11.72 to 14.08)	13.60 (12.38 to 14.82)	14.47 (13.22 to 15.72)
GDP per capital (10,000 Yuan)	4.34 (3.61 to 5.06)	4.70 (3.94 to 5.47)	5.07 (4.26 to 5.88)	5.31 (4.46 to 6.17)	5.56 (4.62 to 6.51)	6.13 (5.14 to 7.13)
Proportion of illiterate population aged 15 and above, %	5.98 (3.77 to 8.18)	5.99 (3.40 to 8.58)	6.22 (3.72 to 8.71)	6.77 (4.35 to 9.18)	6.45 (3.89 to 9.02)	4.71 (3.07 to 6.36)
Registered urban unemployment rate, %	3.32 (3.09 to 3.56)	3.31 (3.07 to 3.56)	3.28 (3.04 to 3.52)	3.26 (3.02 to 3.51)	3.26 (3.01 to 3.50)	3.18 (2.95 to 3.42)
Penetration rate of sanitary toilets in rural areas, %	71.21 (65.62 to 76.80)	73.60 (68.11 to 79.09)	75.20 (69.82 to 80.58)	77.89 (73.03 to 82.74)	79.43 (74.78 to 84.07)	80.76 (76.04 to 85.48)

Table 2 reported the results of fixed effects linear regression models. All MCH indicators showed significantly negative association with the supply of PCP in model 1, 2 and 3 (*p* < 0.05). In addition, the magnitude of primary care indicator coefficient increased each time other variables were added. In model 3, the increase of one PCP per 10,000 population, on average, MMR, LBW, and PMR would decrease 5.260 per 100,000 live births (*p* < 0.001; 95% CI: − 6.745 to − 3.774), 0.106% (*p* < 0.05; 95% CI: − 0.189 to − 0.023) and 0.419 per 1000 live births (*p* < 0.05; 95% CI: − 0.564 to − 0.273) respectively while other variables were held constant. In the sensitivity test, the 1 year lagged primary care indicator had a consistently significant and negative association with each MCH indicator (Table 3), confirming the robustness of results. Besides, the magnitude of association between the supply of PCP and LBW was slightly higher, suggesting a temporal relation between the development of primary care and reduction in LBW.

**Table 2:** Regression model results of MMR, LBW and PMR for the 31 provinces of China, 2012–2017

Variable	MMR			LBW			PMR		
	Model 1	Model 2	Model 3	Model 1	Model 2	Model 3	Model 1	Model 2	Model 3
Primary care physicians per 10,000 population	−4.981*** (− 6.223 to − 3.739)	−5.079*** (− 6.451 to − 3.707)	−5.260*** (− 6.745 to − 3.774)	−0.073* (− 0.144 to − 0.003)	−0.106** (− 0.174 to − 0.019)	−0.106** (− 0.189 to − 0.023)	−0.298*** (− 0.428 to − 0.169)	−0.298** (− 0.441 to − 0.155)	−0.419** (− 0.564 to − 0.273)
Specialty care physicians per 10,000 population	–	0.400 (− 1.939 to 2.740)	−0.124 (− 2.554 to 2.306)	–	0.095 (− 0.037 to 0.227)	0.053 (− 0.083 to 0.189)	–	− 0.001 (− 0.245 to 0.243)	−0.041 (− 0.279 to 0.197)
GDP per capital (10,000 Yuan)	–	–	2.069 (− 0.147 to 4.285)	–	–	0.156* (0.033 to 0.280)	–	–	0.310** (0.093 to 0.527)
Proportion of illiterate population aged 15 and above, %	–	–	−0.176 (− 0.980 to 0.628)	–	–	− 0.006 (− 0.051 to 0.038)	–	–	− 0.163*** (− 0.242 to − 0.084)
Registered urban unemployment rate, %	–	–	− 1.468 (− 5.662 to 2.726)	–	–	− 0.141 (− 0.376 to 0.093)	–	–	− 0.041 (− 0.451 to 0.370)
Penetration rate of sanitary toilets in rural areas, %	–	–	0.087 (− 0.209 to 0.382)	–	–	0.009 (− 0.008 to 0.025)	–	–	0.007 (− 0.022 to 0.036)
Constant	96.389*** (76.333 to 116.446)	92.178*** (60.396 to 123.960)	88.487*** (45.129 to 131.844)	4.15*** (3.006 to 5.287)	3.15** (1.350 to 4.940)	2.725* (0.303 to 5.146)	10.18*** (8.092 to 12.272)	10.20*** (6.881 to 13.508)	11.168*** (6.925 to 15.411)
Observations	186	186	186	186	186	186	186	186	186
Number of provinces	31	31	31	31	31	31	31	31	31
R-squared (within)	0.355	0.356	0.372	0.296	0.305	0.338	0.560	0.560	0.623

*p < 0.05 **p < 0.01 ***p < 0.001. Year fixed effects not shown. 95% CI was in the parentheses

**Table 3:** Sensitivity test for regression models of MMR, LBW and PMR for the 31 provinces of China, 2012–2017

Variable	MMR	LBW	PMR
One year lagged primary care physicians per 10,000 population	−2.303*** (− 3.876 to − 0.730)	− 0.199*** (− 0.303 to − 0.095)	− 0.149*** (− 0.279 to − 0.019)
Specialty care physicians per 10,000 population	−0.411 (− 2.828 to 2.006)	− 0.029 (− 0.189 to 0.131)	0.015 (− 0.185 to 0.214)
GDP per capital (10,000 Yuan)	0.799 (−1.501 to 3.099)	0.224*** (0.071 to 0.376)	0.310** (0.120 to 0.500)
Proportion of illiterate population aged 15 and above, %	−3.197*** (− 4.167 to − 2.227)	− 0.175*** (− 0.239 to − 0.110)	− 0.107** (− 0.187 to − 0.026)
Registered urban unemployment rate, %	0.723 (− 3.136 to 4.582)	−0.026 (− 0.281 to 0.229)	0.128 (− 0.191 to 0.447)
Penetration rate of sanitary toilets in rural areas, %	0.139 (− 0.178 to 0.456)	0.011 (− 0.010 to 0.032)	0.020 (− 0.006 to 0.046)
Constant	60.760*** (19.647 to 101.874)	5.545*** (2.827 to 8.263)	4.494*** (1.097 to 7.891)
Observations	155	155	155
Number of provinces	31	31	31
R-squared (within)	0.399	0.478	0.644

*p < 0.05 **p < 0.01 ***p < 0.001. Year fixed effects not shown. 95% CI was in the parentheses

Table 4 presented analyses stratified by regions and only the results of model 3 were showed due to the limited space. In the western region, the supply of PCP was negatively and significantly (*p* < 0.001) associated with an apparently higher magnitude of MMR (*β =* − 9.007; 95% CI: − 11.639 to − 6.375), LBW (*β =* − 0.210; 95% CI: − 0.380 to − 0.041), and PMR (*β =* − 0.691; 95% CI: − 0.946 to − 0.436), compared with the results of the whole country. In the central and eastern region, the supply of PCP was negatively associated with both MMR and PMR, but only the PMR in central region remained statistically significant (*p* < 0.01). As for the LBW, the direction of effect was opposite but statistically insignificant.

**Table 4:** Regression model results of MMR, LBW and PMR among the western, central, and eastern region of China, 2012–2017

Variable	Western			Central			Eastern		
	MMR	LBW	PMR	MMR	LBW	PMR	MMR	LBW	PMR
Primary care physicians per 10,000 population	−9.007*** (−11.639 to −6.375)	−0.210* (−0.380 to −0.041)	−0.691*** (− 0.946 to − 0.436)	−1.324 (−3.948 to 1.301)	0.142 (− 0.107 to 0.390)	−0.584** (− 1.004 to − 0.164)	−1.359 (− 2.871 to 0.152)	0.067 (− 0.005 to 0.140)	−0.110 (− 0.279 to 0.059)
Specialty care physicians per 10,000 population	0.219 (− 5.776 to 6.214)	0.068 (− 0.318 to 0.455)	−0.364 (− 0.945 to 0.217)	−0.503 (− 3.368 to 2.363)	−0.007 (− 0.279 to 0.264)	0.435 (− 0.024 to 0.894)	1.601 (− 0.569 to 3.771)	−0.056 (− 0.160 to 0.048)	0.150 (− 0.093 to 0.392)
GDP per capital (10,000 Yuan)	−1.941 (− 10.308 to 6.427)	0.103 (− 0.436 to 0.642)	−0.389 (− 1.200 to 0.422)	−1.837 (− 6.861 to 3.187)	0.051 (− 0.425 to 0.527)	0.481 (− 0.324 to 1.286)	−0.742 (− 2.635 to 1.151)	0.095* (0.004 to 0.185)	0.187 (− 0.024 to 0.399)
Proportion of illiterate population aged 15 and above, %	−1.698* (− 3.063 to − 0.333)	−0.020 (− 0.108 to 0.068)	−0.284*** (− 0.416 to − 0.152)	−0.047 (− 1.844 to 1.751)	−0.069 (− 0.239 to 0.101)	−0.015 (− 0.303 to 0.273)	0.994 (− 0.973 to 2.961)	−0.003 (− 0.097 to 0.091)	0.053 (− 0.167 to 0.273)
Registered urban unemployment rate, %	−7.817 (− 17.674 to 2.041)	−0.305 (− 0.940 to 0.330)	−0.146 (− 1.102 to 0.809)	−3.868 (− 7.965 to 0.230)	−0.145 (− 0.533 to 0.243)	0.031 (− 0.626 to 0.687)	−0.724 (− 5.935 to 4.487)	0.152 (− 0.097 to 0.401)	0.279 (− 0.304 to 0.861)
Penetration rate of sanitary toilets in rural areas, %	0.177 (− 0.299 to 0.653)	0.014 (− 0.016 to 0.045)	−0.005 (− 0.051 to 0.041)	−0.148 (− 0.486 to 0.190)	−0.010 (− 0.042 to 0.022)	−0.015 (− 0.069 to 0.040)	−0.106 (− 0.550 to 0.338)	−0.009 (− 0.030 to 0.012)	0.003 (− 0.046 to 0.053)
Constant	218.322*** (127.057 to 309.587)	5.417 (− 0.463 to 11.298)	29.298*** (− 2.343 to 2.680)	74.347** (34.456 to 114.239)	1.464 (− 2.316 to 5.244)	6.908* (0.517 to 13.299)	17.264 (− 48.502 to 83.031)	2.916 (− 0.228 to 6.060)	0.161 (− 7.182 to 7.514)
Observations	72	72	72	48	48	48	66	66	66
Number of provinces	12	12	12	8	8	8	11	11	11
R-squared (within)	0.640	0.317	0.712	0.329	0.534	0.791	0.203	0.819	0.790

*p < 0.05 **p < 0.01 ***p < 0.001. Year fixed effects not shown. 95% CI was in the parentheses

## Discussion

Since limited studies have investigated the association of PCP supply with MCH outcomes in China, this study was intended to fill the gap by conducting an ecological study using a province-level panel dataset. We found that greater PCP supply was associated with improved MCH outcomes and this association was exceptionally significant in the less-developed western region. These findings suggest that PCP supply may have positive effects on improving MCH outcomes and reducing geographical health disparities, which are especially meaningful in China since the contribution of PCPs are somehow neglected and the regional health inequities are still great.

The increase of PCPs was found significantly associated with the decrease of MMR, LBW, PMR in this study, suggesting the contribution of PCPs on improving MCH outcomes in China. The findings are consistent with previous studies in the US [27], England [36], Brazil [37], and Colombia [38]. A literature review that analyzed 36 peer-review studies indicated that the buck of evidence for the effectiveness of primary care was focused on infant and child health [6]. There were also evidences that primary care programs exert positive effects on MCH outcomes in low- and middle-income countries [39, 40]. A qualitative study in China pointed out that community-based interventions and screening for pregnancy complications through PCPs may reduce MMR [41].

Previous studies have explained that primary care may improve MCH outcomes through some mechanisms. As most studies hold, primary care professionals are good at dealing with a range of MCH problems by providing early and continuous prenatal care services such as health education on lifestyle, childbirth education and counseling, and community support and engagement [27]. The shared characteristics of primary care programs with long-term MCH impact were summarized as: providing comprehensive related services, having strong community-based programs, developing strong collaborations with the communities [42]. The mechanisms above consistently reflected that the core functions of primary care such as continuity, comprehensiveness and community-based services may work when explained its positive effects.

In China, the national policy of safeguarding the health of women and children plays a key role when explaining the contributory mechanism of primary care. China has been focusing on improving the health of women and children since 1999, and dedicating to providing services like preventive, curative, protective, rehabilitative, health education, and family planning services. The national program “Reducing Maternal Mortality and Eliminating Neonatal Tetanus” which started in 2000 should be considered as one of the most ambitious national public health interventions in China and globally [25]. At the same time, the government has strengthened the capacity of PCPs by improving the professionalism of MCH workers, working out the professional standardization, and the guidance of maternal and child health care hospitals. MCH management was provided to all pregnant women, newborns, and children as major BPHS item in late 2009, services including premarital and preconception care, prenatal care, child care (ages 0–6), family planning, pregnancy health management, and follow-ups before and after delivery. The government subsidy for BPHS raised from ¥15 per person per year in 2009 to ¥55 in 2018 [43]. All these policies ensured the comprehensiveness, availability, and affordability of health care and thus improved MCH outcomes.

The association between PCP supply and MCH outcomes varied in the eastern, central and western regions which were in different levels of economic development. The western China has lower GDP per capita, higher female illiteracy, and diverse ethnic races, and reported conspicuous effect of PCP supply. While in the more developed eastern and central regions, the positive effects decreased and weakened. This potential contribution of PCP supply on reducing regional health inequities was consistent with researches in other countries. In Brazil, the significant regional differences in the effects of “Family Health Program” expansion suggested that this primary care program has contributed to the reduction of regional inequalities in infant mortality [44]. An ecological study from Colombia indicated that the implementation of primary health care strategy may have contribution to the reductions of the inequality associated with socioeconomic status and living conditions in four child health outcome indicators [45]. Starfield et al. reviewed plenty of existing studies which measured the strength of primary care differently in US and confirmed that stronger primary care system was associated with relatively greater effects on several health outcomes in disadvantaged areas with high levels of income inequality [5]. Previous studies confirmed that the less developed regions have greater absolute levels of contextual risk factors of health which are in urgent need of primary care intervention [46]. The vulnerable population (i.e., the minorities and the poor) also benefited more from a stronger primary care system [8, 9], which may be explained by financial and geographical accessibility, and the comprehensiveness of primary care [38, 47, 48].

The health care systems midwives/nurses are also responsible for much of the care of pregnant women. But unexpectedly the analysis result didn’t meet expectations, there is no significant association between the supply of primary care nurses and MCH outcomes. This may be caused by the different context of China. In China, the PCPs are at the heart of the primary care system while nurses are mainly engaged in carrying out physician’s orders and the number of nurses is allocated according to the number of physicians. Services closely related to maternal and child health care, such as premarital and preconception care, prenatal care, pregnancy health management, and follow-ups before delivery and after discharge are mainly provided by the general practitioner team which are leaded by the physicians while nurses work as assistants. As for midwives, most of them work in the obstetrics and gynecology department of the hospital since the majority of women give birth in hospitals while not in home in the healthcare delivery system in China. The main duty of midwives in China is to assist obstetricians in delivering babies while they are not able to do this independently.

According to the statistical data, we have reached the goal of 2.2 general practitioners per 10,000 population by 2018, but it is still far below the level of most OECD countries (9.26 in France, 7.43 in UK, 7.29 in Germany, data from 2017) [49]. Besides, general practitioners account for only 8.56% of all medical physicians in 2018, while in the developed countries such as UK and US, the proportion is generally 30% ~ 40%. The shortage of PCPs will undermine the quality and value of health services they provide, which is one of the biggest challenges facing primary care strengthening in China. Furthermore, although great achievements have been made in MCH outcomes in China, the gaps between western and eastern regions are still large.

Based on accumulated evidences for the positive effects of PCP supply on MCH outcomes and the critical shortage of primary care workforce, relevant policies should be applied to expand the PCPs. One of the most important initiatives is to establish financial incentive and guarantee mechanisms to raise their salary, and give priority to PCPs to eliminating the persistent payment disparities between primary care and procedural specialties. Besides, the social status of PCPs as well as their sense of professional identity should be improved. With all these endeavors, more excellent and competent physicians would like to stay at the primary care institutions. The Chinese government should also continue to improve the system of general practice medical education in colleges and universities, increase the enrollment of general practice medical students, and train targeted medical professionals for primary care institutions. To exert more influence in reducing regional disparities, incentives should be taken to encourage PCPs to work in less developed regions where their services are most needed.

This research has several limitations. First, this is an ecological study, so it is unable to explore the causality between the included variables, and the results may not be generalized to the individual level due to ecological fallacy. Second, there remains the possibility for latent and unmeasured variable confounding the apparent relationship. Third, the indicators related to the core functions of primary care were not available due to data limitation, which eliminated the explanation of contributory mechanism of primary care. Future studies should not only focus on the supply of PCP but also the effectiveness. More representative and comprehensive indicators should also be explored to measure the strength and quality of primary care.

## Conclusions

To our knowledge, this study is one of the few studies to explore the association between PCP supply and MCH outcomes using nationally-representative panel data in China. It provided new empirical evidence of the potential beneficial impact of PCP supply on improving MCH outcomes and reducing regional disparities. In order to maximize the health-improving and equity-enhancing potential of PCPs, national efforts should be made directing at increasing the PCP supply as well as improving their professional skills, especially in the less developed regions.

## Data Availability

The datasets generated and analyzed during the current study are publicly available in the National Bureau of Statistics in China repository (http://data.stats.gov.cn/, only can be accessed under the Chinese network) and China Statistical Yearbook on Health and Family Planning 2013–2018 repository. (http://navi.cnki.net/KNavi/YearbookDetail?pcode=CYFD&pykm=YSIFE&bh=N2012090077).

## Abbreviations

BPHS: Basic Public Health Services
MCH: Maternal and child health
MMR: Maternal mortality rate
LBW: Low birth weight
PMR: Perinatal mortality rate
PCP: Primary care physician
